# Using Machine Learning to Predict the Diagnosis, Management and Severity of Pediatric Appendicitis

**DOI:** 10.3389/fped.2021.662183

**Published:** 2021-04-29

**Authors:** Ricards Marcinkevics, Patricia Reis Wolfertstetter, Sven Wellmann, Christian Knorr, Julia E. Vogt

**Affiliations:** ^1^Department of Computer Science, ETH Zurich, Zurich, Switzerland; ^2^Department of Pediatric Surgery and Pediatric Orthopedics, Hospital St. Hedwig of the Order of St. John of God, University Children's Hospital Regensburg (KUNO), Regensburg, Germany; ^3^Division of Neonatology, Hospital St. Hedwig of the Order of St. John of God, University Children's Hospital Regensburg (KUNO), University of Regensburg, Regensburg, Germany

**Keywords:** appendicitis, pediatrics, predictive medicine, machine learning, classification

## Abstract

**Background:** Given the absence of consolidated and standardized international guidelines for managing pediatric appendicitis and the few strictly data-driven studies in this specific, we investigated the use of machine learning (ML) classifiers for predicting the diagnosis, management and severity of appendicitis in children.

**Materials and Methods:** Predictive models were developed and validated on a dataset acquired from 430 children and adolescents aged 0-18 years, based on a range of information encompassing history, clinical examination, laboratory parameters, and abdominal ultrasonography. Logistic regression, random forests, and gradient boosting machines were used for predicting the three target variables.

**Results:** A random forest classifier achieved areas under the precision-recall curve of 0.94, 0.92, and 0.70, respectively, for the diagnosis, management, and severity of appendicitis. We identified smaller subsets of 6, 17, and 18 predictors for each of targets that sufficed to achieve the same performance as the model based on the full set of 38 variables. We used these findings to develop the user-friendly online Appendicitis Prediction Tool for children with suspected appendicitis.

**Discussion:** This pilot study considered the most extensive set of predictor and target variables to date and is the first to simultaneously predict all three targets in children: diagnosis, management, and severity. Moreover, this study presents the first ML model for appendicitis that was deployed as an open access easy-to-use online tool.

**Conclusion:** ML algorithms help to overcome the diagnostic and management challenges posed by appendicitis in children and pave the way toward a more personalized approach to medical decision-making. Further validation studies are needed to develop a finished clinical decision support system.

## Introduction

Appendicitis is among the commonest childhood diseases, accounting for a third of admissions for abdominal pain (1). Life-time risk ranges from 6 to 9%, and incidence is highest between 10 and 19 years of age (2). Perforation rates are significantly higher in preschool children than in older children or adults (3).

Diagnosis remains essentially clinical, backed by laboratory data and imaging. In a pooled analysis of serum biomarkers for diagnosing acute appendicitis and perforation, Acharya et al. reported areas under the receiver operating characteristic (AUROC) of 0.75 and 0.69, respectively, for the white blood cell (WBC) count and 0.80 and 0.78 for C-reactive protein (CRP) (4). Despite increasing research there remains no specific biomarker for predicting acute appendicitis in clinical practice (4, 5). Abdominal and, specifically, appendix ultrasonography (US) is the standard imaging modality in children, being low-cost, non-invasive and repeatable, but it remains operator-dependent. Reported sensitivities and specificities for US-based diagnosis range widely: from 87 to 100%, and from 15 to 95% (6). The scores most frequently used to assist physicians in risk-stratifying children with abdominal pain are the Alvarado Score (AS) and Pediatric Appendicitis Score (PAS) (Supplementary Table 1) (7, 8). They may help to exclude appendicitis in an emergency setting (AUROC 0.84 for AS ≤ 3 and PAS ≤ 2) (9), but neither is in widespread routine use.

There are still no consistent and widely used international guidelines for managing acute appendicitis in children. Minimally invasive appendectomy remains the standard treatment of acute appendicitis despite increasing evidence of similar results being achieved by conservative therapy with antibiotics (10, 11), not to mention the reports of spontaneous resolution in uncomplicated cases suggesting that an antibiotic-free approach might be effective in selected school-age children (1, 12).

Machine learning (ML) enhances the early detection and monitoring of multiple medical conditions (13). Supervised learning models leverage large amounts of labeled data to extract complex statistical patterns predictive of a target variable, often achieving superhuman performance levels (14). In this study we applied ML to achieve three outcomes: diagnosing appendicitis in children with abdominal pain; guiding management (conservative without antibiotics vs. operative); and risk stratifying severity (gangrene and perforation). Our aim was to develop and validate a pilot ML tool to support physicians in diagnosing appendicitis at presentation, assessing severity, and deciding management. The purpose of this paper is not to develop a finished clinical decision support system, but rather to present a pilot study for a promising research prototype based on machine learning. To the best of our knowledge, this is the first study using ML to simultaneously predict diagnosis, conservative vs. operative management, and severity in children with suspected appendicitis.

## Materials and Methods

### Data Acquisition

The cohort study included all children and adolescents aged 0-18 years admitted with abdominal pain and suspected appendicitis to the Department of Pediatric Surgery at the tertiary Children's Hospital St. Hedwig in Regensburg, Germany, over the 3-year period from January 1, 2016 to December 31, 2018. Non-inclusion criteria were prior appendectomy, abdominal conditions such as chronic inflammatory bowel disease or intestinal duplication, simultaneous appendectomy, and treatment with antibiotics for concurrent disease such as pneumonia, resulting in a final total of 430 patients (Table 1). The study was approved by the University of Regensburg institutional review board (no. 18-1063-101) which also waived informed consent to routine data analysis. For patients followed up after discharge, informed consent was obtained from parents or legal representatives. All methods were performed in accordance with the relevant guidelines and regulations. Conservative management was defined as intravenous fluids, enemas, analgesics, and clinical/US monitoring without antibiotics in an inpatient setting. For patients with criteria for simple appendicitis presenting clinical and sonographic improvement, non-operative therapy was maintained, otherwise they underwent operation. Appendectomy was laparoscopic in 88% of cases and traditional in 12%. Histological and intra-operative findings were assessed. The routine procedure for children and adolescents with suspected appendicitis is summarized in Supplementary Figure 1.

**Table 1:** Counts of patients in different diagnosis, management, and severity categories.

	**Appendicitis: Uncomplicated/Complicated**	**No appendicitis: Uncomplicated/Complicated**	**Total: Uncomplicated/Complicated**
Surgical management:	114/51	0/0	114/51
Conservative management:	82/0	183/0	265/0
Total:	196/51	183/0	379/51

*Rows correspond to different management categories; columns correspond to different diagnoses. Each cell contains counts of patients with uncomplicated appendicitis or without appendicitis and with complicated appendicitis (separated by “/”) in the corresponding subgroup*.

### Data Description

Our analysis considered predictive models for three binary response variables:

- diagnosis: appendicitis (*n* = 247, 57.21%) and no appendicitis (*n* = 183, 42.79%)
- management: surgical (*n* = 165, 38.37%) and conservative (*n* = 265, 61.63%)
- severity: complicated (*n* = 51, 11.86%) and uncomplicated appendicitis or no appendicitis (*n* = 379, 88.14%).

The “appendicitis” category included both acute and subacute cases, while “surgical” comprised primary and secondary surgical treatment. It is important to note that we could not confirm the diagnosis in every patient: histology was only possible in patients who underwent surgery. Conservatively treated patients were retrospectively assigned the “appendicitis” label only if they had AS and/or PAS values ≥ 4 and an appendix diameter ≥ 6 mm. Diagnosis was a proxy for confirmed disease status. Patients with the above criteria for appendicitis who were first treated conservatively (*n* = 86) were contacted at least 6 months after discharge (mean 28 months). We reached 61 individuals, five of whom had since undergone appendectomy and were therefore included in the surgical group. Appendicitis was classified as “uncomplicated” in all conservatively treated cases. The “uncomplicated” category also included patients without appendicitis since none had complications during treatment; it was almost 8 times larger than the “complicated” category. To address this major imbalance, we investigated the use of cost-sensitive classification models, e.g., by introducing prior category probabilities in random forest models (15), but performance was not markedly improved. The other two category pairs were reasonably balanced. Table 1 contains detailed counts of patients within different diagnosis, management, and severity categories.

Our analysis considered 38 predictor variables including patient and US data. Variables were continuous, binary, and categorical. All were measured before treatment was assigned and none represent intraoperative findings. Supplementary Table 2 contains explanations of all 38 predictor variables included in the model development and validation.

We computed summary statistics for patient subgroups, based on the three responses. Statistical tests for differences between subgroups were performed in the R programming language (version 3.6.2) (16). Summary and test statistics were based on non-missing data only. Chi-squared tests of independence were used for discrete variables and unpaired two-sided Mann-Whitney *U*-tests for continuous variables; *p*-values were adjusted for multiple comparisons using Hommel's method (17). A level of α = 0.05 was chosen for statistical significance. Predictors with several categories were binarized prior to the chi-squared test.

### Preprocessing

The dataset contained missing values. As a preprocessing step, we performed missing data imputation using the *k*-nearest neighbors (*k*-NN) (with *k* = 5) method based on Gower distance (18), as implemented in the R *VIM* package (19). This method imputes missing variables in every instance based on values occurring within the proximity given by Gower distance for continuous, categorical, and ordered variables (19). To avoid data leakage and the introduction of spurious associations between predictor and response variables, we performed the imputation without response variables and separately for train and test sets.

### Machine Learning

To predict the above response variables, we trained and validated three different ML models for classification in the R programming language (version 3.6.2) (16):

- logistic regression (LR), as implemented in the R *glmnet* package (20);
- random forest (RF) (21), as implemented in the R *randomForest* package (15);
- generalized boosted regression model (GBM) (22), as implemented in the R *gbm* package (23).

LR is only capable of learning a linear decision boundary to differentiate between classes, whereas the RF and GBM models are non-linear ensemble classification methods and can thus potentially learn more complex patterns. Both RF and GBM achieve this by training many simple classifiers and consequently aggregating their predictions into a single estimate.

To identify which variables were crucial for predictive performance, we compared classifiers trained on the following predictor subsets:

- full set of 38 predictor variables
- without US data (“US-free”)
- without the “peritonitis/abdominal guarding” variable
- without US data or the “peritonitis/abdominal guarding” variable.

It was interesting to investigate whether responses could be predicted without including the US variables that might be operator-dependent or unavailable in emergencies (24, 25). We singled out the “peritonitis/abdominal guarding” variable because detection can be unreliable, requiring an experienced examiner; our analysis considered it under three subcategories: (i) no peritonitis/abdominal guarding, (ii) localized, and (iii) generalized.

#### Evaluation Metrics

**Figure 1:**
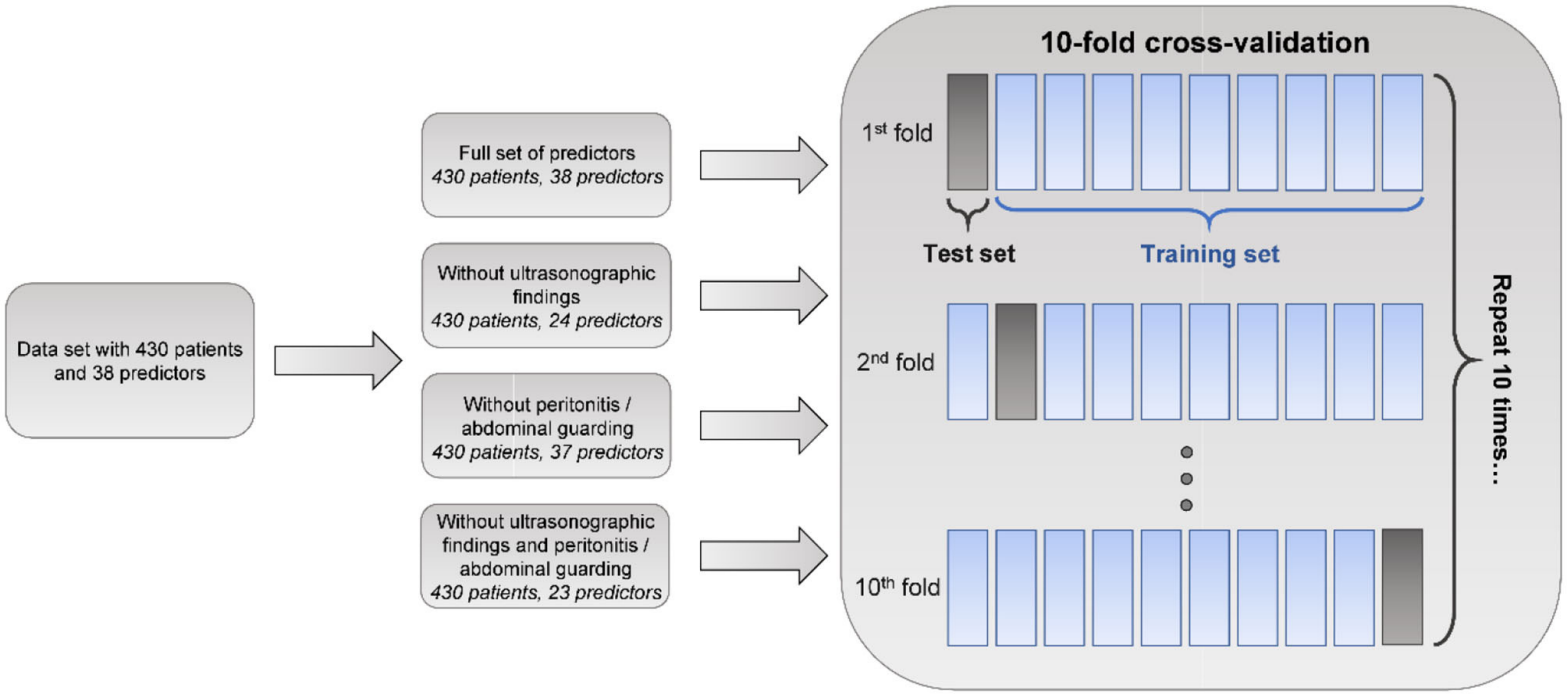
Machine learning analysis schematic. Machine learning models, namely logistic regression (LR), random forest (RF), and generalized boosted regression model (GBM), based on various sets of predictor variables, are evaluated using areas under receiver operating characteristic (AUROC) and precision-recall (AUPR) curves in the 10-fold cross-validation procedure. Ten-fold cross-validation is a standard procedure for evaluating the performance of predictive ML models wherein the model is trained on 90% of the data and tested on the remaining 10% repeatedly for 10 disjoint test folds. In our analysis, missing value imputation was performed separately for train and test sets using the *k*-nearest neighbors (*k*-NN) method.

To evaluate and compare predictive models, we performed 10-fold cross-validation (CV) (26), using the *k*-NN method for imputing missing values separately for train and test sets. Ten-fold cross-validation is a standard procedure for the evaluation of ML models, wherein the model is repeatedly trained on 90% of data and tested on 10% of withheld data for 10 disjoint test folds. Predictive performance was assessed using AUROC and area under the precision-recall (AUPR) curve (Figure 1) (27). AUPR is particularly informative for classification problems with extreme class imbalance (27). It was therefore more appropriate for comparing models predicting appendicitis severity. We compared model performance using two-sided 10-fold cross-validated paired *t*-tests at a significance level α = 0.05 (28). In addition to AUROC and AUPR, sensitivity, specificity, negative and postive predictive values of the classifiers were evaluated.

#### Variable Selection

In a clinical setting, variables can be systemically missing at test time. We therefore also examined the importance of predictor variables in case the number of predictors used by classifiers could be reduced without compromising their performance. Both RF and GBM provide measures of variable importance (15, 21, 23). We examined the averages of class-specific measures of variable importance given by the mean decrease in RF accuracy (15). We trained random forests on 300 bootstrap resamples of the data and used boxplots to visualize the distributions of the importance values obtained (29).

In addition, we cross-validated a variable selection procedure based on the RF importance measure to determine the minimal number of variables that could be used without compromising predictive performance. The procedure can be summarized as follows. For number of predictors *q* from 1 to 38, repeat:

1. Train full RF model $M_{full}$ (all predictor variables included) on the train set. Retrieve variable importance values.
2. Train RF model $M_{q}$ based on *q* predictors with the highest importance values, on the train set.
3. Evaluate AUROC and AUPR of $M_{q}$ on the test set.
4. Repeat steps 1-3 for all 10 folds in CV.

This procedure evaluates the performance of random forest classifiers that use varying numbers of predictors chosen on the basis of importance values.

Finally, we examined which variable subsets were chosen consistently, for each *q*. For *q* from 1 through 38, we trained random forest classifiers on 300 bootstrap resamples of the data and counted how many times each predictor was among the *q* most important variables. In this way, we could assess the variability of a set of *q* most important predictors, rather than provide a single selection which could be unstable because based on only one replication of the experiment.

## Results

Distributions of several predictors differed significantly (at level α = 0.05) for all three responses, namely, AS, PAS, appendix diameter, body temperature, WBC count, neutrophil percentage, CRP, and peritonitis/abdominal guarding. These variables had previously been identified as useful in predicting appendicitis (8, 30–32). Table 2 and Supplementary Tables 3, 4 show the summary statistics and statistical test results for patient subgroups based on response variables. In general, the descriptive statistics suggested that the data featured strong associations between some predictors and responses.

**Table 2:** Dataset description for patients with and without appendicitis.

**Variable**	**Appendicitis (*n* = 247)**	**No appendicitis(*n* = 183)**	***P*-value**
Age, years	11.48 [9.18, 13.29]	12.10 [9.57, 14.46]	0.6
Male sex, %	58.13	47.83	0.5
Height, cm	149.1 [137.5, 162.0]	152.2 [139.6, 164.0]	0.8
Weight, kg	39.75 [31.00, 52.75]	47.10 [32.48, 57.08]	0.4
Body mass index (BMI), kg/m^2^	17.84 [15.72, 20.55]	18.90 [15.95, 22.39]	0.3
Alvarado score, pts	7 [5, 8]	4 [3, 6]	**≤0.001**
Pediatric appendicitis score, pts	5 [4, 7]	4 [3, 5]	**≤0.001**
Peritonitis/abdominal guarding, %	61.38	7.61	**≤0.001**
Migration of pain, %	30.89	18.48	0.09
Tenderness in right lower quadrant (RLQ), %	97.97	95.63	1.0
Rebound tenderness, %	40.98	25.68	**≤0.05**
Cough tenderness, %	32.65	19.57	0.06
Psoas sign, %	27.85	33.91	1.0
Nauseous/vomiting, %	62.20	48.37	0.1
Anorexia, %	31.71	25.68	1.0
Body temperature, °C	37.75 [37.20, 38.20]	37.20 [36.80, 37.85]	**≤0.001**
Dysuria, %	3.45	7.82	0.7
Abnormal stool, %	28.40	27.07	1.0
White blood cell count, 10^3^/μl	13.80 [10.68, 17.40]	8.80 [7.00, 11.90]	**≤0.001**
Neutrophils, %	78.95 [70.40, 84.17]	61.50 [52.35, 77.55]	**≤0.001**
C-reactive protein, mg/l	15.00 [4.00, 46.00]	1.00 [0.00, 13.00]	**≤0.001**
Ketones in urine, %	44.94	31.54	0.5
Erythrocytes in urine, %	23.42	20.81	1.0
White blood cells in urine, %	12.03	12.75	1.0
Visibility of appendix, %	86.53	34.97	**≤0.001**
Appendix diameter, mm	8.00 [7.00, 10.00]	5.00 [4.05, 5.28]	**≤0.001**
Free intraperitoneal fluid, %	52.56	31.84	**≤0.01**
Irregular appendix layers, %	41.74	11.11	0.1
Target sign, %	67.37	9.10	**≤0.001**
Appendix perfusion, %	74.47	12.50	**≤0.05**
Surrounding tissue reaction, %	86.01	16.22	**≤0.001**
Pathological lymph nodes, %	62.20	74.70	0.8
Mesenteric lymphadenitis, %	79.69	81.08	1.0
Thickening of the bowel wall, %	55.77	19.44	**≤0.05**
Ileus, %	25.00	0.00	0.17
Coprostasis, %	34.15	42.42	1.0
Meteorism, %	59.18	84.48	0.1
Enteritis, %	16.67	69.57	**≤0.05**

*Distributions of variables are presented as either medians with interquartile ranges (in square brackets) or percentages. For significant differences, p-values are reported in bold as “≤0.001,” “≤0.01” or “≤0.05” (at significance level α = 0.05)*.

Table 3 shows the 10-fold CV results for the different ML classifiers for predicting diagnosis, management, and severity. For diagnosis classification, full RF (average AUROC: 0.96, average AUPR: 0.94) and GBM (average AUROC: 0.96, average AUPR: 0.94) models significantly outperformed logistic regression (average AUROC: 0.91, average AUPR: 0.88). AUROC and AUPR *p*-values were 0.002 and 0.006 for RF, and 0.007 and 0.03 for GBM. This suggests benefit from using non-linear classification methods for predicting a diagnosis of appendicitis. The full GBM and RF classifiers performed equally with respect to both evaluation metrics. All ML models performed considerably better than the random classifier, that is, a random guess. On average, classifiers that used the full set of predictors had higher AUROCs and AUPRs than the clinical baselines, such as AS, PAS, and suspected diagnosis, given by hospital specialists. Based on the CV results, US input is crucial for accurately diagnosing appendicitis because average AUROC and AUPR degraded in all models when it was absent. Peritonitis had less influence on prediction quality.

**Table 3:** Ten-fold cross-validation results for logistic regression (LR), random forest (RF), and generalized boosted regression (GBM) models for predicting diagnosis, management, and severity.

**Classifier**	**Diagnosis**		**Management**		**Severity**	
	**AUROC (±SD)**	**AUPR (±SD)**	**AUROC (±SD)**	**AUPR (±SD)**	**AUROC (±SD)**	**AUPR (±SD)**
Random	0.50	0.43	0.50	0.38	0.50	0.12
AS	0.75	0.71	—	—	—	—
PAS	0.71	0.67	—	—	—	—
AS or PAS ≥ 4 and appendix diameter ≥ 6 mm	0.79	0.83	—	—	—	—
Suspected diagnosis	0.73	0.85	—	—	—	—
LR (full)	0.91 (±0.04)	0.88 (±0.07)	0.90 (±0.04)	0.88 (±0.06)	0.82 (±0.13)	0.53 (±0.26)
LR (w/o US)	0.82 (±0.06)	0.71 (±0.12)	0.91 (±0.04)	0.90 (±0.05)	**0.91 (±0.09)**	0.69 (±0.26)
LR (w/o peritonitis/abdominal guarding)	0.90 (±0.04)	0.87 (±0.06)	0.83 (±0.04)	0.79 (±0.06)	0.82 (±0.15)	0.58 (±0.28)
LR (w/o US and peritonitis/abdominal guarding)	0.77 (±0.06)	0.67 (±0.14)	0.80 (±0.04)	0.77 (±0.06)	0.81 (±0.16)	0.62 (±0.26)
RF (full)	**0.96 (±0.01)**	**0.94 (±0.03)**	**0.94 (±0.02)**	0.92 (±0.05)	0.90 (±0.08)	**0.70 (±0.17)**
RF (w/o US)	0.85 (±0.05)	0.77 (±0.11)	0.93 (±0.03)	0.90 (±0.07)	0.90 (±0.08)	0.67 (±0.18)
RF (w/o peritonitis/abdominal guarding)	0.95 (±0.01)	0.93 (±0.05)	0.85 (±0.07)	0.79 (±0.11)	0.88 (±0.10)	0.65 (±0.23)
RF (w/o US and peritonitis/abdominal guarding)	0.80 (±0.06)	0.73 (±0.11)	0.78 (±0.05)	0.70 (±0.10)	0.86 (±0.10)	0.58 (±0.21)
GBM (full)	**0.96 (±0.02)**	**0.94 (±0.03)**	**0.94 (±0.02)**	**0.93 (±0.04)**	0.90 (±0.07)	0.64 (±0.21)
GBM (w/o US)	0.85 (±0.06)	0.75 (±0.10)	0.92 (±0.04)	0.90 (±0.05)	**0.91 (±0.07)**	0.60 (±0.25)
GBM (w/o peritonitis/abdominal guarding)	0.95 (±0.02)	0.92 (±0.05)	0.87 (±0.05)	0.82 (±0.08)	0.84 (±0.13)	0.58 (±0.25)
GBM (w/o US and peritonitis/abdominal guarding)	0.79 (±0.06)	0.71 (±0.11)	0.79 (±0.07)	0.72 (±0.08)	0.84 (±0.12)	0.55 (±0.27)

*Results are given by average areas under receiver operating characteristic (AUROC) and precision-recall (AUPR) curves and standard deviations across 10 folds. “Full” models use all predictors; models “w/o US” were trained without ultrasonographic findings; models “w/o peritonitis/abdominal guarding” were trained without “peritonitis/abdominal guarding” predictor; and models “w/o US and peritonitis/abdominal guarding” were trained without ultrasonographic findings and “peritonitis/abdominal guarding” predictor. For fixed classification rules, such as Alvarado (AS) and pediatric appendicitis scores (PAS), AUROC and AUPR on the whole dataset are reported without standard deviations. For random classifiers, we report expected AUROC and AUPR. “Random” corresponds to a random guess and serves as a naïve baseline. Bold values correspond to the best average performances achieved across all models*.

For predicting management, the full RF and GBM models had the highest average AUROC (0.94), while the full GBM had the highest average AUPR (0.93). Both non-linear methods significantly outperformed logistic regression (average AUROC: 0.90, average AUPR: 0.88). AUROC and AUPR *p*-values were 0.01 and 0.06 (non-significant) for RF, and 0.02 and 0.03 for GBM. All models had considerably better average AUROCs and AUPRs than the random classifier. Based on the CV results, peritonitis is a very important variable for predicting management. Average model performance dropped considerably when removing this variable. US findings did not affect prediction quality as much as when diagnosing appendicitis.

As for appendicitis severity, US-free logistic regression achieved the highest average AUROC (0.91) alongside US-free GBM, while full-set RF achieved the highest average AUPR (0.70) (Table 3). Although all models performed considerably better than the random classifier, complicated appendicitis appeared harder to predict than either diagnosis or management. The AUPRs were much lower, and all models had high variances across the folds. This could be due to the very low prevalence of complicated appendicitis (12% of all patients). There was little gain in performance from using non-linear classification methods. The differences in AUROC and AUPR between RF, GBM, and (US-free) logistic regression were non-significant. AUROC and AUPR *p*-values were 0.94 and 0.97 for RF, and 0.76 and 0.58 for GBM. US input had almost no effect on average classifier performance whereas peritonitis was important and its exclusion markedly decreased AUROC and AUPR values in all models.

We also evaluated model sensitivities, specificities, and negative and positive predictive values (NPV/PPV). Tables 4, 5 contain results of the 10-fold CV for all three responses. In this analysis, a threshold of 0.5 was used to predict labels. When incorporating any of these models into clinical decision-making, the threshold will have to be chosen based on the desired sensitivity and specificity. For diagnosis, full non-linear classifiers achieved better combinations of sensitivity, specificity, NPV, and PPV than the clinical baseline (AS or PAS ≥ 4 and appendix diameter ≥ 6 mm). Similar to the evaluation in Table 3, on average, non-linear classifiers performed noticeably better than logistic regression in predicting diagnosis.

**Table 4:** Ten-fold cross-validation results for logistic regression (LR), random forest (RF), and generalized boosted regression (GBM) models for predicting diagnosis, management, and severity.

**Classifier**	**Diagnosis**		**Management**		**Severity**	
	**Sens. (±SD)**	**Spec. (±SD)**	**Sens. (±SD)**	**Spec. (±SD)**	**Sens. (±SD)**	**Spec. (±SD)**
Random	0.57	0.43	0.62	0.38	0.88	0.12
AS or PAS ≥ 4 and appendix diameter ≥ 6 mm	0.91	0.73	—	—	—	—
Suspected diagnosis	**1.00**	0.46	—	—	—	—
LR (full)	0.88 (±0.06)	0.76 (±0.11)	0.85 (±0.09)	0.82 (±0.09)	0.93 (±0.05)	0.42 (±0.32)
LR (w/o US)	0.75 (±0.06)	0.72 (±0.09)	0.92 (±0.07)	0.85 (±0.05)	0.95 (±0.04)	**0.52 (±0.29)**
LR (w/o peritonitis/abdominal guarding)	0.87 (±0.07)	0.76 (±0.12)	0.84 (±0.10)	0.68 (±0.15)	0.94 (±0.05)	0.40 (±0.36)
LR (w/o US and peritonitis/abdominal guarding)	0.77 (±0.06)	0.67 (±0.11)	0.82 (±0.06)	0.63 (±0.07)	0.97 (±0.05)	0.44 (±0.34)
RF (full)	0.91 (±0.03)	0.86 (±0.08)	**0.94 (±0.07)**	0.80 (±0.09)	**0.98 (±0.02)**	0.45 (±0.16)
RF (w/o US)	0.81 (±0.07)	0.71 (±0.07)	0.93 (±0.07)	0.82 (±0.07)	0.97 (±0.02)	0.44 (±0.13)
RF (w/o peritonitis/abdominal guarding)	0.91 (±0.04)	**0.90 (±0.06)**	0.86 (±0.07)	0.65 (±0.18)	**0.98 (±0.02)**	0.37 (±0.17)
RF (w/o US and peritonitis/abdominal guarding)	0.79 (±0.06)	0.64 (±0.11)	0.81 (±0.06)	0.56 (±0.06)	0.98 (±0.02)	0.40 (±0.15)
GBM (full)	0.93 (±0.02)	0.86 (±0.07)	0.93 (±0.07)	**0.86 (±0.07)**	0.97 (±0.02)	0.46 (±0.18)
GBM (w/o US)	0.80 (±0.07)	0.74 (±0.11)	0.91 (±0.08)	0.85 (±0.05)	0.97 (±0.03)	0.44 (±0.16)
GBM (w/o peritonitis/abdominal guarding)	0.92 (±0.04)	0.83 (±0.09)	0.88 (±0.04)	0.66 (±0.11)	0.97 (±0.03)	0.47 (±0.20)
GBM (w/o US and peritonitis/abdominal guarding)	0.80 (±0.06)	0.61 (±0.10)	0.82 (±0.07)	0.59 (±0.09)	0.97 (±0.03)	0.47 (±0.19)

*Results are given by average sensitivities (sens.) and specificities (spec.) with standard deviations across 10 folds. “Full” models use all predictors; models “w/o US” were trained without ultrasonographic findings; models “w/o peritonitis/abdominal guarding” were trained without the “peritonitis/abdominal guarding” predictor; and models “w/o US and peritonitis/abdominal guarding” were trained without ultrasonographic findings or the “peritonitis/abdominal guarding” predictor. For all classifiers, a probability threshold of 0.5 was used to differentiate between classes. “Random” corresponds to a random guess and serves as a naïve baseline. Bold values correspond to the best average performances achieved across all models*.

**Table 5:** Ten-fold cross-validation results for logistic regression (LR), random forest (RF), and generalized boosted regression (GBM) models for predicting diagnosis, management, and severity.

**Classifier**	**Diagnosis**		**Management**		**Severity**	
	**PPV (±SD)**	**NPV (±SD)**	**PPV (±SD)**	**NPV (±SD)**	**PPV (±SD)**	**NPV (±SD)**
Random	0.57	0.43	0.62	0.38	0.88	0.12
AS or PAS ≥ 4 and appendix diameter ≥ 6 mm	0.82	0.85	—	—	—	—
Suspected diagnosis	0.71	**1.00**	—	—	—	—
LR (full)	0.83 (±0.07)	0.83 (±0.09)	0.89 (±0.06)	0.79 (±0.09)	0.92 (±0.04)	0.51 (±0.28)
LR (w/o US)	0.78 (±0.08)	0.68 (±0.10)	**0.91 (±0.03)**	0.88 (±0.10)	**0.94 (±0.04)**	0.61 (±0.34)
LR (w/o peritonitis/abdominal guarding)	0.83 (±0.09)	0.82 (±0.11)	0.82 (±0.05)	0.74 (±0.09)	0.92 (±0.04)	0.45 (±0.29)
LR (w/o US and peritonitis/abdominal guarding)	0.76 (±0.09)	0.68 (±0.10)	0.78 (±0.04)	0.68 (±0.09)	0.93 (±0.04)	0.69 (±0.33)
RF (full)	0.89 (±0.08)	0.88 (±0.05)	0.88 (±0.04)	**0.90 (±0.12)**	0.93 (±0.03)	**0.80 (±0.26)**
RF (w/o US)	0.78 (±0.07)	0.74 (±0.10)	0.89 (±0.04)	0.88 (±0.10)	0.93 (±0.03)	0.72 (±0.24)
RF (w/o peritonitis/abdominal guarding)	**0.92 (±0.05)**	0.88 (±0.07)	0.81 (±0.09)	0.74 (±0.13)	0.92 (±0.04)	0.77 (±0.24)
RF (w/o US and peritonitis/abdominal guarding)	0.74 (±0.11)	0.69 (±0.09)	0.75 (±0.05)	0.65 (±0.10)	0.92 (±0.03)	0.72 (±0.23)
GBM (full)	0.89 (±0.07)	0.90 (±0.04)	**0.91 (±0.04)**	0.88 (±0.10)	0.93 (±0.02)	0.67 (±0.21)
GBM (w/o US)	0.81 (±0.09)	0.73 (±0.11)	**0.91 (±0.03)**	0.87 (±0.11)	0.93 (±0.02)	0.70 (±0.25)
GBM (w/o peritonitis/abdominal guarding)	0.87 (±0.08)	0.89 (±0.06)	0.81 (±0.04)	0.77 (±0.08)	0.93 (±0.03)	0.72 (±0.24)
GBM (w/o US and peritonitis/abdominal guarding)	0.73 (±0.09)	0.70 (±0.10)	0.76 (±0.06)	0.67 (±0.11)	0.93 (±0.03)	0.68 (±0.23)

*Results are given by average positive and negative predictive values (PPV/NPV) with standard deviations across 10 folds. “Full” models use all predictors; models “w/o US” were trained without ultrasonographic findings; models “w/o peritonitis/abdominal guarding” were trained without the “peritonitis/abdominal guarding” predictor; and models “w/o US and peritonitis/abdominal guarding” were trained without ultrasonographic findings or the “peritonitis/abdominal guarding” predictor. For all classifiers, a probability threshold of 0.5 was used to differentiate between classes. “Random” corresponds to a random guess and serves as a naïve baseline. Bold values correspond to the best average performances achieved across all models*.

**Figure 2:**
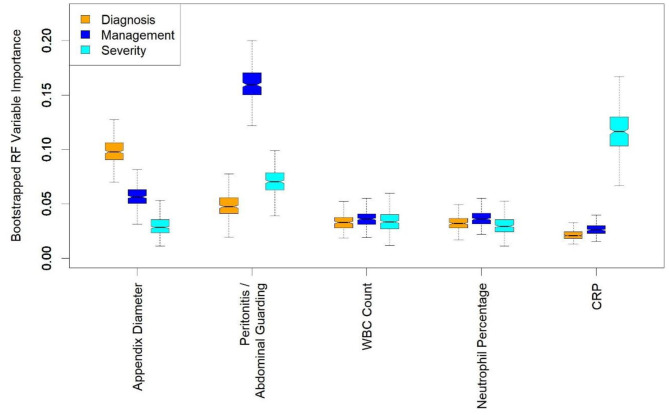
Boxplots of random forest (RF) importance values for a few most important predictors. RF variable importance quantifies how important each variable is for predicting the considered outcome. Appendix diameter, peritonitis/abdominal guarding, white blood cell (WBC) count, neutrophil percentage, and C-reactive protein (CRP) are among 10 most important variables for predicting diagnosis, management and severity. Distributions were obtained by training random forest classifiers on 300 bootstrap resamples of the dataset. The bootstrapping was performed to provide uncertainty about variable importance values, rather than mere point estimates.

To identify the most crucial predictive variables, we trained RF classifiers on 300 bootstrap resamples of the dataset and obtained a distribution of importance values for every predictor. The RF variable importance quantifies how important each variable is for predicting the outcome in the random forest model. For diagnosing appendicitis, on average, the most important predictors were appendix diameter, appendix visibility on US, and peritonitis. For management, they were peritonitis, appendix diameter, and WBC count. For severity, they were CRP, peritonitis, and body temperature (details in Figure 2). Plots of importance values for the full set of predictors are shown in Supplementary Figure 2. Overall, these findings agreed with the statistical results in Table 2 and Supplementary Tables 3, 4. Predictor variables that differ significantly across patient subgroups are often among the most important features used by random forests for predictions.

**Figure 3:**
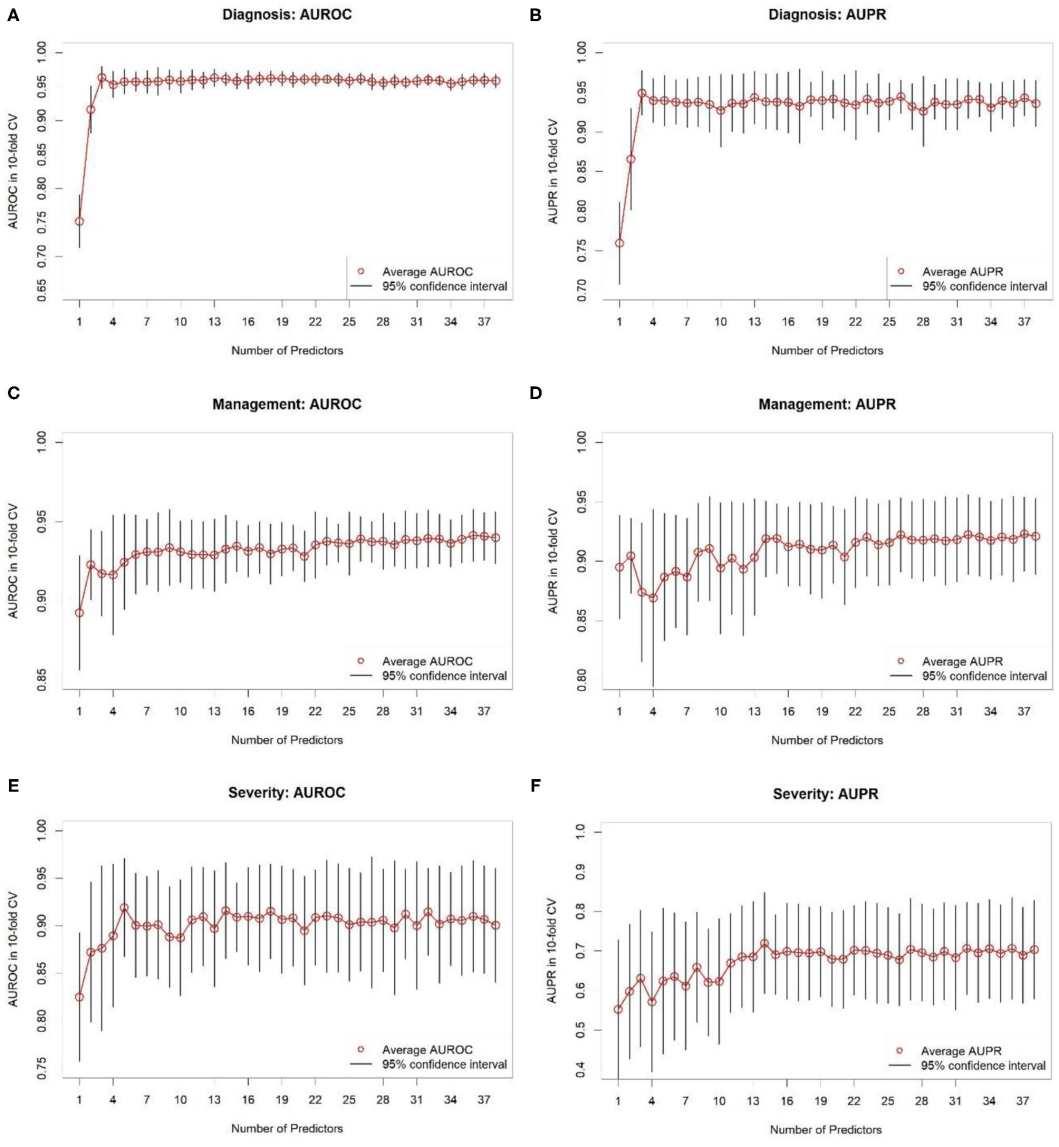
Results of 10-fold cross-validation for random forest classifiers based on different numbers of predictor variables selected based on variable importance. **(A,B)** Show areas under receiver operating characteristic (AUROC) and precision-recall (AUPR) curves, respectively, for predicting diagnosis. **(C,D)** Show AUROCs and AUPRs, respectively, for predicting management. **(E,F)** Show AUROCs and AUPRs, respectively, for predicting severity. Black-colored bars correspond to 95% confidence intervals, constructed using *t*-distribution; red-colored dots correspond to averages. Recall that random classifier AUROCs are 0.50 for all three targets and random classifier AUPRs are 0.43, 0.38, and 0.12 for diagnosis, treatment, and complicated appendicitis, respectively.

In addition, we performed variable selection using RF importance. Figure 3 contains AUROC and AUPR plots for RF models based on varying numbers of predictors. For predicting diagnosis, classifier AUROC and AUPR values saturated at *q* = 3 (Figures 3A,B). Thus, a few variables suffice for accurate appendicitis risk stratification. For management, there was a steady increase in average AUROC (Figure 3C) with an increase in the number of predictor variables selected. For AUPPR, classifiers with <14 predictors (Figure 3D) had higher variances in 10-fold CV. Predictive performance stabilized at *q* = 14. Similarly, for predicting severity, average AUROC and AUPR increased steadily with model complexity (Figures 3E,F). AUROC saturated at *q* = 5, and AUPR at *q* = 11. For all three prediction tasks, we observed that the full set of predictors is far from necessary because full-model performance levels can be achieved with a smaller number of variables.

We used bootstrapping to determine how frequently variables were selected based on their RF importance. For predicting diagnosis, we looked at choosing *q* = 3 most important variables. The variables chosen in >5% of bootstrap resamples included appendix diameter, appendix visibility on US, peritonitis, target sign, WBC count, and neutrophil percentage. For management we examined a subset of size *q* = 14. The variables selected in ≥5% of bootstrap resamples included peritonitis, CRP, neutrophil percentage, WBC count, appendix diameter, enteritis, target sign, appendix perfusion, AS, body temperature, age, surrounding tissue reaction, appendix layer structure, weight, body mass index (BMI), height, and PAS. For severity we chose a subset of *q* = 11 variables. The following predictors were selected in >5% of bootstrap resamples: peritonitis, CRP, body temperature, WBC count, neutrophil percentage, appendix diameter, appendix perfusion, weight, age, bowel wall thickening, height, AS, BMI, ileus, appendix layer structure, PAS, erythrocytes in urine, and target sign. Supplementary Table 5 summarizes these variable selection results.

## Online Tool

We provide an easy-to-use online tool for the three response variables at http://papt.inf.ethz.ch/ (33). The RF models implemented in this tool use limited sets of predictors chosen based on variable importance and 10-fold CV. We chose random forests because they outperformed logistic regression and were, in general, on a par with GBM. We included the variables selected into subsets in ≥5% of bootstrap resamples of the dataset. The tool presents a pilot status and was developed for educational use only. Even in further steps after prospective validation, practical clinical considerations must be incorporated into decision-making.

## Discussion

This observational study of children referred with abdominal pain to the pediatric surgical department used different ML models to predict the diagnosis, management and severity of appendicitis. Starting with a granular dataset including demographic, clinical, laboratory, and US variables, we identified a minimal subset of key predictors and trained classifiers that far outperformed conventional scores such as the AS and PAS. Since all the variables we used in this study are standardized and widely available for evaluating patients with abdominal pain, our findings are broadly relevant. We also developed the Appendicitis Prediction Tool (APT) to predict the diagnosis, management and severity of appendicitis with unlimited online access.

A basic challenge with ML models is that their performance depends largely on the quality and representativity of the training data, and their applicability in real life depends on the accessibility of required features (34). For example, assessing abdominal guarding as a sign of peritonitis can be challenging during initial presentation of small children with abdominal pain. If this finding is unclear, it is recommended that assessment be repeated during the clinical observation period, if necessary under analgesia (35, 36). Based on RF variable importance and CV results, we found that “peritonitis/abdominal guarding” had the highest importance for predicting management, but not appendicitis or appendicitis severity, for which other predictors were more important (Figure 2). The AS and PAS can be easily calculated after clinical examination and hemogram. Although abdominal and appendix US is the most suitable and cost-effective imaging modality for suspected appendicitis, it is highly operator-dependent, requiring years of training, particularly for children, and is not always on hand in every ED. That is why we also trained models without “peritonitis/abdominal guarding,” without US, and without either “peritonitis/abdominal guarding” or US. These variables are not mandatory in the prediction tool, making it easier to deploy. The predictors are imputed using the *k*-NN method if the user decides to omit them. Nevertheless, based on the CV results (Table 3), the models incorporating US variables performed considerably better in predicting diagnosis and management and hence are preferred, to avoid complications and misdiagnosis. Most children with missed appendicitis on presenting to the ED of a tertiary care hospital did not undergo US (67 vs. 13% of correctly diagnosed cases, *p* < 0.05) (37).

Several studies have used ML to support the diagnosis of appendicitis (30, 38, 39). Four recent studies have focused exclusively on the pediatric population (40–43). Reismann et al. performed feature selection and trained a logistic regression to diagnose appendicitis and differentiate between uncomplicated and complicated cases of pediatric acute appendicitis (40). They analyzed laboratory variables and appendix diameter in US and achieved AUROCs of 0.91 and 0.80 for diagnosing appendicitis and differentiating complicated appendicitis, respectively. Akmese et al. analyzed demographic and laboratory data and used a range of ML methods to predict whether pediatric patients with suspected acute appendicitis underwent surgery (41). In their analysis, gradient boosting attained the highest accuracy (95%). Similar to Akmese et al. (41) Aydin et al. detected pediatric appendicitis based on demographic and pre-operative laboratory data (42). In addition, they differentiated between complicated and uncomplicated appendicitis. Their decision tree model achieved AUROCs of 0.94 and 0.79 for predicting appendicitis and uncomplicated appendicitis, respectively. Stiel et al. applied different appendicitis scores (AS, PAS, Heidelberg, and Tzanakis Score) to a dataset of pediatric patients presenting with abdominal pain to predict diagnosis and perforated appendicitis (43). The Heidelberg Score was modified and a data-driven score was developed using decision trees and random forests, achieving AUROCs of, respectively, 0.92 and 0.86 for appendicitis diagnosis, and both 0.71 for perforation.

Our own analysis focused exclusively on the pediatric population given the particularities of appendicitis in this age range: atypical clinical course and elevated perforation rates in preschool-aged children, high prevalence, and multiple differential diagnoses (44, 45). In addition to demographic, laboratory, and ultrasonographic data, we considered clinical predictors, such as peritonitis/abdominal guarding, and appendicitis scores (AS and PAS). Moreover, we targeted the prediction of all three targets simultaneously: diagnosis, management, and severity. None of the machine learning models mentioned above were deployed as an open access online tool (40–43), whereas our models are available as an easy-to-use APT.

Our 10-fold CV results (Table 3) are overall comparable to the performance levels reported by Reismann et al. (40), Akmese et al. (41), Aydin et al. (42), and Stiel et al. (43) whose studies are similar to ours. Compared to the previous work on using ML to predict pediatric appendicitis (40–43), our analysis considers the most extensive set of variables and, to the best of our knowledge, is the first to simultaneously predict diagnosis, management, and severity of appendicitis in pediatric patients. In a retrospective study Cohen et al. found that children with a normal WBC count and an appendix non-visualized on US could initially be kept under observation (46). According to our data, appendix visibility on US is one of the most important predictors for diagnosis (Figure 2).

In the presented collective, pediatric patients with suspected simple appendicitis and persistent symptoms after initial treatment and evaluation at the ED were admitted to further observation and therapy, as shown in Supplementary Figure 1. They received initial clinical support, e.g., intravenous fluids, enemas, without antibiotics. Eighty two patients with clinical and US signs of uncomplicated appendicitis showed clinical improvement, including appendicitis regression signs in US. Therefore, they were discharged after a period of observation. Several studies indicate that simple and complicated appendicitis might have a different pathophysiology, suggesting that some forms of uncomplicated appendicitis may be reversible, and, as an alternative to operation, could be treated with or even without antibiotics (1, 47–50). Ohba et al. (12) conducted a prospective study of pediatric appendicitis based on US findings such as appendix diameter, wall structure, and perfusion. Their results support the possibility of treating pediatric patients conservatively without antibiotics if abundant blood flow in the appendix submucosal layer is still detectable.

The APT is an academic instrument whose sensitivity and specificity require further clinical testing. This prototype was developed based on our first dataset as a pilot trial with a promising application of ML as a basis for further prospective studies. It needs a larger training dataset and external blinded validation before it can be integrated into clinical decision-making. The model could be extended to differentiate patients requiring primary surgery from those suitable for conservative management with or without antibiotics by identifying the characteristics supporting spontaneous regression of acute appendicitis. Furthermore, predictive models could be used to support the decision on which surgical approach is the best suitable for the patient. Certain minimal invasive approaches such as TULAA (trans-umbilical laparoscopic-assisted appendectomy) may benefit from preoperative patient stratification, guiding the decision between single incision vs. 2-trocar technique (51).

## Strengths and Limitations

The current dataset was acquired from patients admitted to a pediatric surgical unit with suspected appendicitis. Those with mild symptoms and/or rapid improvement had already been discharged by the emergency department. This can be assumed to have increased the probability of appendicitis among surgical admissions. The predictors for all three outcomes include clinical, laboratory, and US parameters that are readily and cost-effectively available during a patient's work-up. Limitations include certain missing variables, a limited number of patients, especially with complicated appendicitis, the lack of a definitive histological diagnosis in conservatively managed patients (we provide a more detailed discussion of this limitation in the Supplementary Material), and the current absence of external validation. Due to these limitations, the APT is merely a research prototype and must not be relied on for health or personal advice.

## Conclusion

Pediatric appendicitis remains an important disease with a heterogeneous presentation. The APT should help clinicians identify and manage patients with potential appendicitis. It could become an important tool for clinical observation in the near future. The goal of further research should be the expanded application of ML models for the early differential diagnosis of children with abdominal pain. We see it as a valuable tool for recognizing appendicitis severity and facilitating a personalized management approach.

## Data Availability Statement

The dataset analyzed is available in anonymized form alongside with the code in a GitHub repository: https://github.com/i6092467/pediatric-appendicitis-ml.

## Ethics Statement

The study involving human participants was reviewed and approved by the University of Regensburg institutional review board (Ethikkommission der Universität Regensburg, no. 18-1063-101), which also waived informed consent to routine data analysis. For patients followed up after discharge, written informed consent was obtained from parents or legal representatives.

## Author Contributions

All authors made substantial contributions to conception and design, analyses and interpretation of data, and revising the article. PRW and CK performed clinical data acquisition, coordination and check. PRW performed literature review and contributed to the manuscript. RM performed statistical and machine learning analysis and contributed to the manuscript. CK and SW supervised the clinical part of the project. JV supervised the machine learning part of the project. All authors have read the manuscript and approved its submission.

## Conflict of Interest

The authors declare that the research was conducted in the absence of any commercial or financial relationships that could be construed as a potential conflict of interest.
