# Supplementary material for: Using Machine Learning to Predict the Diagnosis, Management and Severity of Pediatric Appendicitis
Source: Front Pediatr. 2021 Apr 29;9:662183. doi: 10.3389/fped.2021.662183 (PMC8116489; doi:10.3389/fped.2021.662183)
Supplement: Supplementary file 1 [file Data_Sheet_1.docx]

Supplementary Material

**Supplementary Tables**

**Table S1.** Appendicitis scores: Alvarado score (AS) and Pediatric appendicitis score (PAS).

| **Alvarado Score (AS)** | | **Pediatric Appendicitis Score (PAS)** | |
| --- | --- | --- | --- |
| **Diagnostic criteria** | **Value** | **Diagnostic criteria** | **Value** |
| Migration of pain | 1 | Migration of pain | 1 |
| Anorexia / ketones in urine | 1 | Anorexia | 1 |
| Nausea / emesis | 1 | Nausea / emesis | 1 |
| Tenderness in right lower quadrant | 2 | Tenderness in right lower quadrant | 2 |
| Rebound pain | 1 | Cough / percussion / hopping tenderness | 2 |
| Elevation of temperature (≥ 37.3 °C) | 1 | Fever (≥ 38 °C) | 1 |
| Leukocytosis (> 10,000/µl) | 2 | Leukocytosis (> 10,000/µl) | 1 |
| Neutrophilia (> 75%) | 1 | Neutrophilia (> 75%) | 1 |
| **Total** | **10** | **Total** | **10** |

**Table S2**. Explanation of predictor variables included in multivariate predictive models for diagnosis, management, and severity of appendicitis. The table reports how and when each variable was measured and states variable types along with categories (where applicable).

| **Variable** | **Explanation** | **Mode & Time of Measurement** | **Variable Type & Values** |
| --- | --- | --- | --- |
| **Demographic** | | | |
| Age, years | Obtained from date of birth | At hospital admission | Continuous |
| Sex | Registered gender | At hospital admission | Binary: female / male |
| Height, cm | Patient’s height | At hospital admission | Continuous |
| Weight, kg | The amount patient weighs | At hospital admission | Continuous |
| Body mass index (BMI), kg/m^2^ | Measures body fat; patient's weight divided by the square of the height | At hospital admission | Continuous |
| **Appendicitis Scores** | | | |
| Alvarado score (AS), pts | See Table S1 | At hospital admission, after clinical examination and laboratory data | Discrete |
| Pediatric appendicitis score (PAS), pts | See Table S1 | At hospital admission, after clinical examination and laboratory data | Discrete |
| **Clinical** | | | |
| Peritonitis/abdominal guarding | Spasm of abdominal wall muscles detected on palpation, usually a result of inflammation | At hospital admission, during clinical examination, or after a few hours of observation, if needed after analgesia | Categorical:   - no - localized - generalized |
| Migration of pain | Abdominal pain; usually starts in epigastrium and moves to the right lower quadrant | At hospital admission, during clinical examination or anamnesis | Binary: no / yes |
| Tenderness in right lower quadrant (RLQ) | Right iliac fossa pain detected on palpation | At hospital admission, during clinical examination | Binary: no / yes |
| Rebound tenderness | A state in which pain is felt on the release of pressure over the abdomen | At hospital admission, during clinical examination | Binary: no / yes |
| Cough tenderness | Abdominal pain by forced cough | At hospital admission, during clinical examination | Binary: no / yes |
| Psoas sign | Abdominal pain produced by extension of the hip | At hospital admission, during clinical examination | Binary: negative / positive |
| Nausea/vomiting | Feeling of sickness/ejection of contents from stomach through the mouth | Anamnesis | Binary: no / yes |
| Anorexia | Loss of appetite | Anamnesis | Binary: no / yes |
| Body temperature, °C | Measured by a thermometer placed in the rectum or in the auditory canal | At hospital admission or after a few hours of observation | Continuous |
| Dysuria | Pain or other difficulty during urination | Anamnesis | Binary: no / yes |
| Stool | Characteristics of bowel movements | Anamnesis | Categorical:   - normal - diarrhea - obstipation |
| **Laboratory (from routine blood samples in case of suspected appendicitis)** | | | |
| White blood cell count (WBC), 10^3^/µl | The number of leucocytes in a unit volume of blood; inflammation parameter | At hospital admission, obtained from a routine hemogram | Continuous |
| Neutrophils, % | Mature WBC in the granulocytic series | At hospital admission, obtained from differential WBC | Continuous |
| C-reactive protein (CRP), mg/l | Protein produced by the liver, elevated in case of inflammation, infection, or injury | At hospital admission, obtained from blood sample | Continuous |
| Ketones in urine | Presence of ketone bodies in urine, e.g. in case of anorexia | At hospital admission, obtained from routine urine status | Categorical:   - no - + - ++ - +++ |
| Erythrocytes in urine | Blood in urine | At hospital admission, obtained from routine urine status | Categorical:   - neg: < 5 ery/µl - +: approx. 5-10 ery/µl - ++: approx. 25 ery/µl - +++: approx. 50 ery/µl |
| White blood cells in urine | Leucocytes in urine, e.g., in case of infection | At hospital admission, obtained from routine urine status | Categorical:   - no - + - ++ - +++ |
| **Abdominal Ultrasound** | | | |
| Visibility of appendix | Detectability of the vermiform appendix during sonographic examination | At hospital admission, after clinical examination, or after a few hours of observation | Binary: no / yes |
| Appendix diameter, mm | Maximal outer diameter of the appendix | At hospital admission, after clinical examination, or after a few hours of observation | Continuous |
| Free intraperitoneal fluid | Free fluids inside the abdomen | At hospital admission, after clinical examination, or after a few hours of observation | Binary: no / yes |
| Appendix layer structure | Distribution and characteristics of appendix layers, e.g., irregular in case of an increasing inflammation | At hospital admission, after clinical examination, or after a few hours of observation | Binary: regular / irregular |
| Target sign | Axial image of appendix with a fluid-filled center surrounded by echogenic mucosa and submucosa and hypoechoic muscularis | At hospital admission, after clinical examination, or after a few hours of observation | Binary: no / yes |
| Appendix perfusion | Blood flow to the appendix wall | At hospital admission, after clinical examination, or after a few hours of observation | Categorical:   - unremarkable - hypoperfused - hyperperfused |
| Surrounding tissue reaction | Inflammation signs in tissue (i.a. in omentum/fat tissue) surrounding appendix | At hospital admission, after clinical examination, or after a few hours of observation | Binary: no / yes |
| Pathological lymph nodes | Enlarged and inflamed intra-abdominal lymph nodes | At hospital admission, after clinical examination, or after a few hours of observation | Binary: no / yes |
| Mesenteric lymphadenitis | Collection of enlarged/inflamed lymph nodes in intestinal mesentery | At hospital admission, after clinical examination, or after a few hours of observation | Binary: no / yes |
| Thickening of the bowel wall | Edema of the intestinal wall, > 2-3 mm for small bowel wall thickening | At hospital admission, after clinical examination, or after a few hours of observation | Binary: no / yes |
| Ileus | Sonographic signs of paralytic ileus (e.g., dilated intestinal loops, pendulum peristalsis or absence of peristalsis) | At hospital admission, after clinical examination, or after a few hours of observation | Binary: no / yes |
| Coprostasis | Fecal impaction in the colon | At hospital admission, after clinical examination, or after a few hours of observation | Binary: no / yes |
| Meteorism | Accumulation of gas in the intestine | At hospital admission. after clinical examination, or after a few hours of observation | Binary: no / yes |
| Enteritis | Sonographic features of gastroenteritis, e.g., wall thickening of ileum, increased peristalsis | At hospital admission after clinical examination, or after a few hours of observation | Binary: no / yes |

**Table S3**. Dataset description for patients treated surgically and conservatively. Distributions of variables are presented as either medians with interquartile ranges (in square brackets) or percentages. For significant differences, $p$-values are reported as “$\boldsymbol{\leq}$ **0.001**”, “$\boldsymbol{\leq}$ **0.01**” or “$\boldsymbol{\leq}$ **0.05**” (at significance level $\alpha=0.05$).

| **Variable** | **Surgical  (**$\boldsymbol{n=165}$**)** | **Conservative (**$\boldsymbol{n=265}$**)** | $\boldsymbol{p}$**-value** |
| --- | --- | --- | --- |
| Age, years | 11.36 [8.98, 13.83] | 11.83 [9.58, 14.04] | 0.9 |
| Male sex, % | 57.58 | 51.32 | 0.9 |
| Height, cm | 149.0 [136.0, 162.0] | 151.0 [140.0, 164.0] | 0.9 |
| Weight, kg | 39.50 [30.0, 51.00] | 43.00 [32.50, 56.00] | 0.6 |
| Body mass index (BMI), kg/m^2^ | 17.84 [15.59, 20.44] | 18.63 [15.95, 21.97] | 0.7 |
| Alvarado score, pts | 7 [6, 8] | 5 [4, 6] | $\boldsymbol{\leq}$ **0.001** |
| Pediatric appendicitis score, pts | 6 [4, 7] | 4 [3, 6] | $\boldsymbol{\leq}$ **0.001** |
| Peritonitis/abdominal guarding, % | 86.67 | 8.30 | $\boldsymbol{\leq}$ **0.001** |
| Migration of pain, % | 29.70 | 23.02 | 0.9 |
| Tenderness in right lower quadrant (RLQ), % | 98.18 | 96.21 | 0.9 |
| Rebound tenderness, % | 43.90 | 28.52 | $\boldsymbol{\leq}$ **0.05** |
| Cough tenderness, % | 32.93 | 23.40 | 0.5 |
| Psoas sign, % | 27.08 | 32.53 | 0.9 |
| Nauseous/vomiting, % | 66.67 | 49.81 | $\boldsymbol{\leq}$ **0.05** |
| Anorexia, % | 33.94 | 26.14 | 0.8 |
| Body temperature, °C | 37.80 [37.20, 38.30] | 37.20 [36.80, 38.00] | $\boldsymbol{\leq}$ **0.001** |
| Dysuria, % | 4.55 | 5.84 | 0.9 |
| Abnormal stool, % | 25.45 | 29.28 | 0.9 |
| White blood cell count, 10^3^/µl | 15.35 [12.00, 18.70] | 9.70 [7.53, 13.00] | $\boldsymbol{\leq}$ **0.001** |
| Neutrophils, % | 80.10 [73.85, 85.30] | 67.05 [54.58, 79.67] | $\boldsymbol{\leq}$ **0.001** |
| C-reactive protein, mg/l | 20.00 [5.00, 77.50] | 3.00 [0.00, 16.00] | $\boldsymbol{\leq}$ **0.001** |
| Ketones in urine, % | 52.58 | 31.90 | 0.08 |
| Erythrocytes in urine, % | 24.74 | 20.95 | 0.9 |
| White blood cells in urine, % | 8.25 | 14.29 | 0.9 |
| Visibility of appendix, % | 79.27 | 55.30 | $\boldsymbol{\leq}$ **0.001** |
| Appendix diameter, mm | 9.00 [7.50, 11.00] | 6.10 [5.00, 7.58] | $\boldsymbol{\leq}$ **0.001** |
| Free intraperitoneal fluid, % | 57.52 | 35.38 | $\boldsymbol{\leq}$ **0.001** |
| Irregular appendix layers, % | 62.32 | 10.96 | $\boldsymbol{\leq}$ **0.001** |
| Target sign, % | 80.00 | 23.33 | $\boldsymbol{\leq}$ **0.001** |
| Appendix perfusion, % | 91.67 | 45.16 | $\boldsymbol{\leq}$ **0.001** |
| Surrounding tissue reaction, % | 92.08 | 45.57 | $\boldsymbol{\leq}$ **0.001** |
| Pathological lymph nodes, % | 54.17 | 74.36 | 0.3 |
| Mesenteric lymphadenitis, % | 83.87 | 79.44 | 0.9 |
| Thickening of the bowel wall, % | 64.71 | 25.93 | $\boldsymbol{\leq}$ **0.05** |
| Ileus, % | 38.46 | 0.00 | $\boldsymbol{\leq}$ **0.01** |
| Coprostasis, % | 34.78 | 39.22 | 0.9 |
| Meteorism, % | 62.50 | 77.33 | 0.9 |
| Enteritis, % | 8.33 | 62.07 | 0.1 |

**Table S4**. Dataset description for patients with different severity of appendicitis. Distributions of variables are presented as either medians with interquartile ranges (in square brackets) or percentages. For significant differences, $p$-values are reported as “$\boldsymbol{\leq}$ **0.001**”, “$\boldsymbol{\leq}$ **0.01**” or “$\boldsymbol{\leq}$ **0.05**” (at significance level $\alpha=0.05$).

| **Variable** | **Complicated  (**$\boldsymbol{n=51}$**)** | **Uncomplicated  (**$\boldsymbol{n=379}$**)** | $\boldsymbol{p}$**-value** |
| --- | --- | --- | --- |
| Age, years | 10.90 [7.72, 12.96] | 11.69 [9.39, 13.96] | 1.0 |
| Male sex, % | 50.98 | 54.09 | 1.0 |
| Height, cm | 146.3 [130.0, 162.2] | 150.5 [138.2, 163.0] | 1.0 |
| Weight, kg | 37.00 [26.35, 52.00] | 42.00 [32.00, 56.00] | 1.0 |
| Body mass index (BMI), kg/m^2^ | 17.72 [15.38, 20.57] | 18.23 [15.96, 21.27] | 1.0 |
| Alvarado score, pts | 7 [6, 9] | 6 [4, 7] | $\boldsymbol{\leq}$ **0.001** |
| Pediatric appendicitis score, pts | 6 [5, 8] | 5 [3, 6] | $\boldsymbol{\leq}$ **0.001** |
| Peritonitis/abdominal guarding, % | 98.04 | 30.34 | $\boldsymbol{\leq}$ **0.001** |
| Migration of pain, % | 33.33 | 24.54 | 1.0 |
| Tenderness in right lower quadrant (RLQ), % | 94.12 | 97.35 | 1.0 |
| Rebound tenderness, % | 43.14 | 33.24 | 1.0 |
| Cough tenderness, % | 33.33 | 26.19 | 1.0 |
| Psoas sign, % | 21.95 | 31.53 | 1.0 |
| Nauseous/vomiting, % | 84.31 | 52.51 | $\boldsymbol{\leq}$ **0.001** |
| Anorexia, % | 47.06 | 26.72 | 0.1 |
| Body temperature, °C | 38.30 [37.85, 38.75] | 37.30 [37.00, 38.00] | $\boldsymbol{\leq}$ **0.001** |
| Dysuria, % | 6.38 | 5.22 | 1.0 |
| Abnormal stool, % | 37.50 | 26.60 | 0.5 |
| White blood cell count, 10^3^/µl | 17.10 [12.80, 21.05] | 11.20 [7.95, 15.00] | $\boldsymbol{\leq}$ **0.001** |
| Neutrophils, % | 82.55 [76.92, 85.60] | 72.80 [56.40, 81.50] | $\boldsymbol{\leq}$ **0.001** |
| C-reactive protein, mg/l | 119.0 [30.5, 198.0] | 6.0 [1.0, 21.0] | $\boldsymbol{\leq}$ **0.001** |
| Ketones in urine, % | 65.52 | 35.61 | 0.2 |
| Erythrocytes in urine, % | 48.26 | 19.42 | 0.06 |
| White blood cells in urine, % | 13.79 | 12.23 | 1.0 |
| Visibility of appendix, % | 66.00 | 64.29 | 1.0 |
| Appendix diameter, mm | 10.0 [8.6, 12.0] | 7.0 [5.5, 9.0] | $\boldsymbol{\leq}$ **0.001** |
| Free intraperitoneal fluid, % | 64.44 | 41.03 | 0.1 |
| Irregular appendix layers, % | 76.47 | 30.40 | $\boldsymbol{\leq}$ **0.05** |
| Target sign, % | 66.67 | 43.70 | 1.0 |
| Appendix perfusion, % | 100.00 | 62.00 | 1.0 |
| Surrounding tissue reaction, % | 93.75 | 66.89 | 0.1 |
| Pathological lymph nodes, % | 55.56 | 70.07 | 1.0 |
| Mesenteric lymphadenitis, % | 75.00 | 80.95 | 1.0 |
| Thickening of the bowel wall, % | 71.43 | 35.14 | 0.5 |
| Ileus, % | 72.73 | 3.45 | $\boldsymbol{\leq}$ **0.001** |
| Coprostasis, % | 50.00 | 35.94 | 1.0 |
| Meteorism, % | 72.73 | 72.92 | 1.0 |
| Enteritis, % | 0.00 | 50.00 | 1.0 |

**Table S5**. Variables that were ranked among $q$ most important predictors in random forest (RF) in at least 5% of 300 bootstrap resamples. For predicting diagnosis, we considered a subset of $q=3$ variables; for management, we considered $q=14$; and, for severity, we chose $q=11$.

| **Variable** | **% of bootstrap resamples among** $\boldsymbol{q}$ **most important predictors in RF** | | |
| --- | --- | --- | --- |
|  | **Diagnosis (**$\boldsymbol{q=3}$**)** | **Management (**$\boldsymbol{q=14}$**)** | **Severity (**$\boldsymbol{q=11}$**)** |
| **Demographic** | | | |
| Age, years | < 5.00 | 86.33 | 69.33 |
| Height, cm | < 5.00 | 37.00 | 42.67 |
| Weight, kg | < 5.00 | 74.33 | 72.00 |
| Body mass index (BMI), kg/m^2^ | < 5.00 | 45.00 | 36.67 |
| **Appendicitis Scores** | | | |
| Alvarado score, pts | < 5.00 | 94.67 | 36.67 |
| Pediatric appendicitis score, pts | < 5.00 | 23.00 | 18.67 |
| **Clinical** | | | |
| Peritonitis/abdominal guarding | 66.33 | 100.00 | 100.00 |
| Body temperature, °C | < 5.00 | 89.00 | 100.00 |
| **Laboratory (at entry)** | | | |
| White blood cell count, 10^3^/µl | 6.67 | 100.00 | 99.67 |
| Neutrophils, % | 6.00 | 100.00 | 98.67 |
| C-reactive protein, mg/l | < 5.00 | 100.00 | 100.00 |
| Erythrocytes in urine | < 5.00 | < 5.00 | 12.67 |
| **Ultrasound** | | | |
| Visibility of appendix | 99.67 | < 5.00 | < 5.00 |
| Appendix diameter, mm | 100.00 | 100.00 | 97.33 |
| Appendix layers structure | < 5.00 | 74.33 | 24.33 |
| Target sign | 20.67 | 98.67 | 7.67 |
| Appendix perfusion | < 5.00 | 96.67 | 91.00 |
| Surrounding tissue reaction | < 5.00 | 78.33 | < 5.00 |
| Thickening of the bowel wall | < 5.00 | < 5.00 | 56.67 |
| Ileus | < 5.00 | < 5.00 | 25.33 |
| Enteritis | < 5.00 | 99.00 | < 5.00 |

**Supplementary Figures**


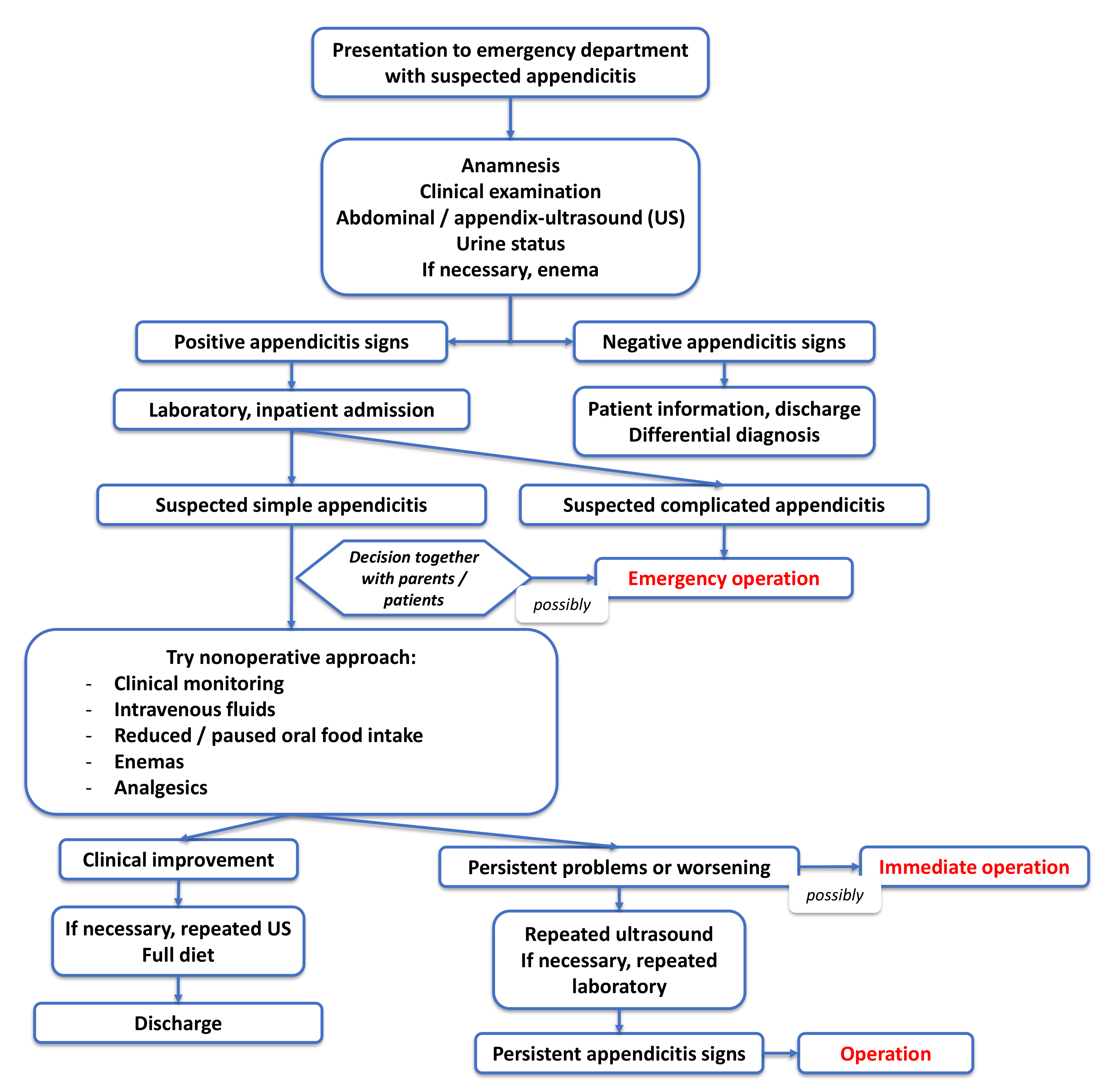


**Figure S1**. Routine patient management procedure schematic. Decision between conservative management and operation was made by a senior pediatric surgeon based upon clinical presentation combined with laboratory and ultrasound findings. Simple appendicitis is defined by phlegmonous appendicitis, or subacute forms (e.g., catarrhal, appendix fibrosis). Complicated appendicitis includes abscess formation, gangrene, or perforation. *From: Department of Pediatric Surgery and Pediatric Orthopedics, Hospital St. Hedwig of the Order of St. John of God, University Children's Hospital Regensburg (KUNO), Regensburg, Germany.*


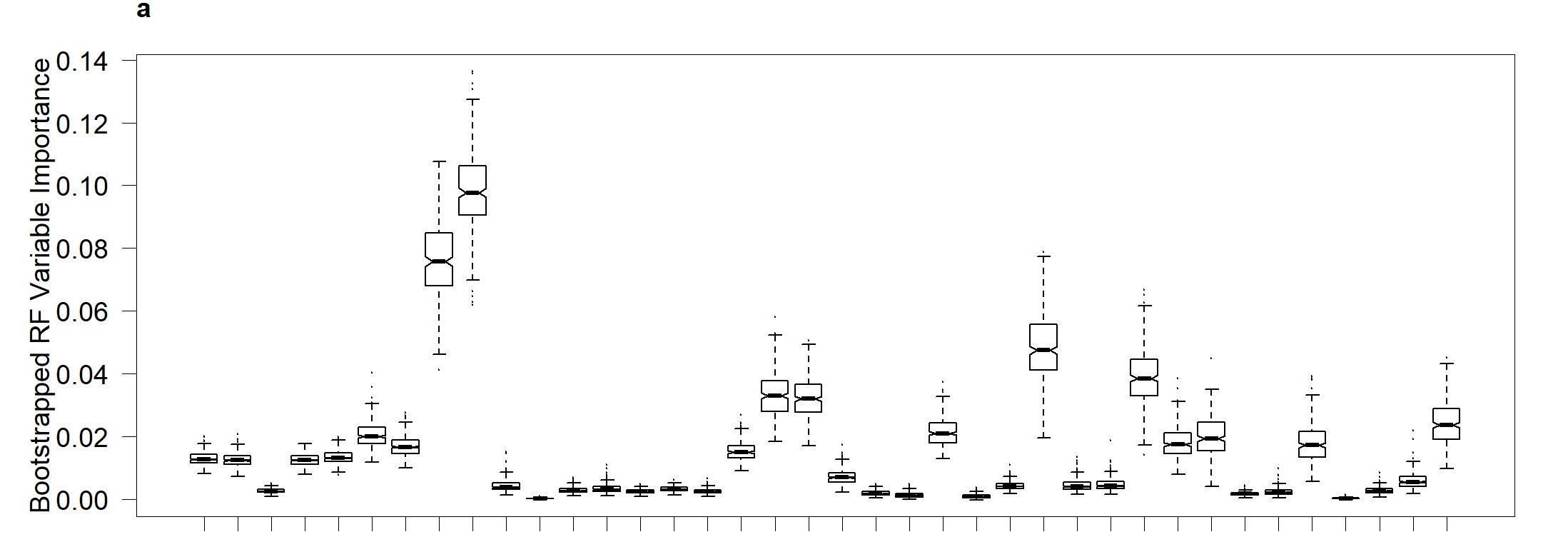

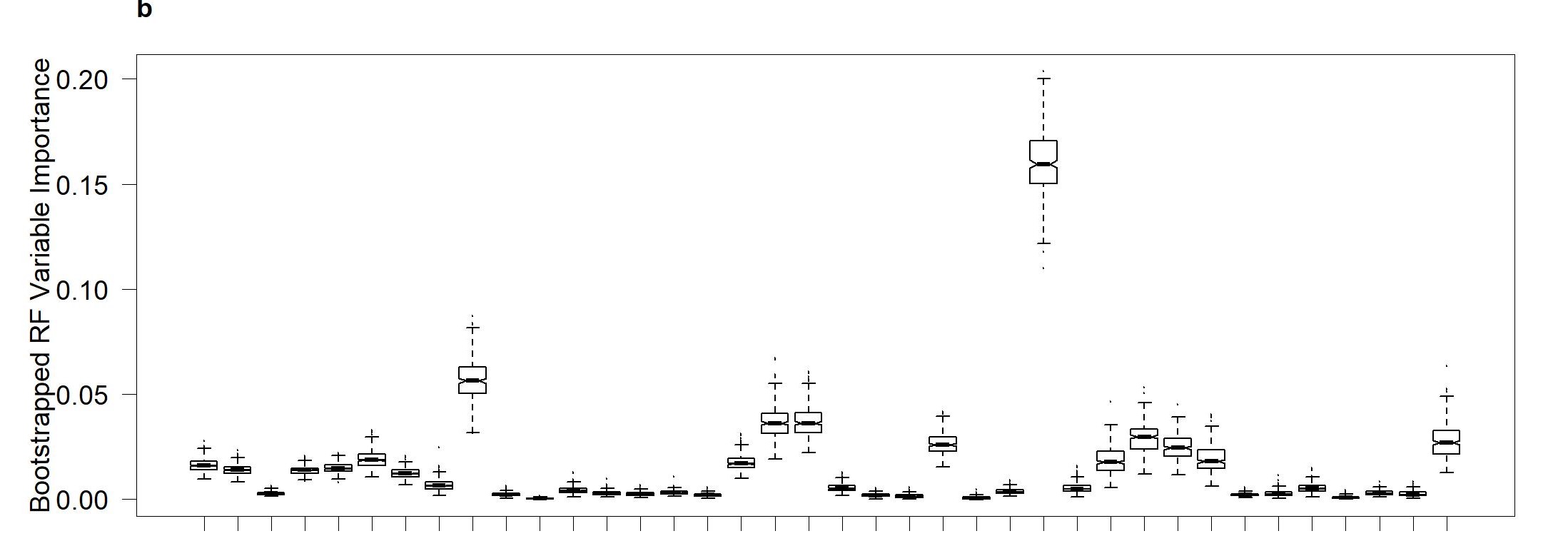

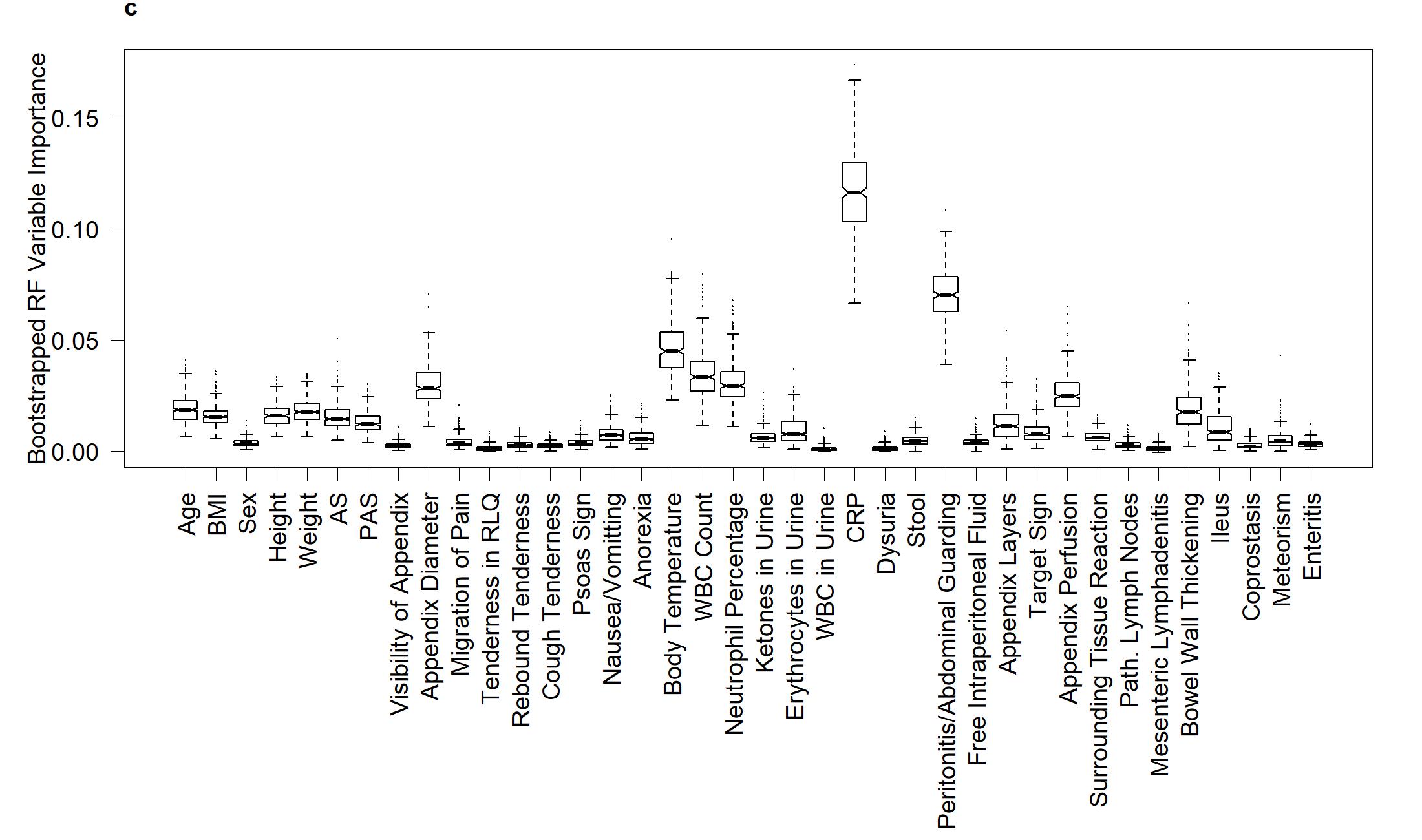


**Figure S2**. Boxplots of random forest (RF) importance values of all variables for predicting **(a)** diagnosis, **(b)** management, and **(c)** severity. Distributions were obtained by training random forest classifiers on 300 bootstrap resamples of the dataset. BMI: body mass index; AS: Alvarado score; PAS: pediatric appendicitis score; RLQ: right lower quadrant; WBC: white blood cell; CRP: C-reactive protein.

**Absence of histologically confirmed diagnoses and its influence on ML models**

We accept that the absence of histological confirmation in many patients is a major limitation of this analysis and we acknowledged as much in our original manuscript (Strengths and Limitations). However, we believe that the lack of confirmed diagnoses does not render the predictive model useless or inadequate. Below we provide the results of an exploratory analysis that should alleviate these concerns.


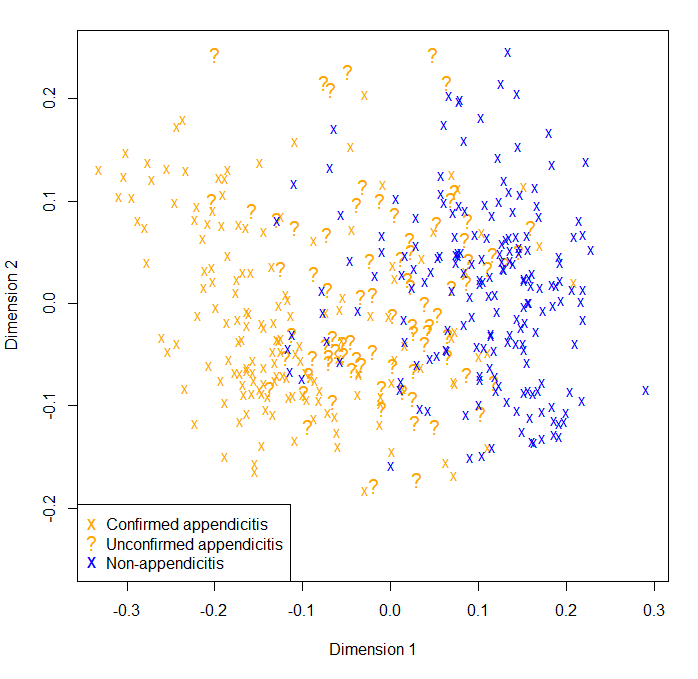
We reduced the data dimensionality (after *k*-NN imputation) by applying multidimensional scaling (MDS) to pairwise Gower distances (NB: dimensionality reduction was *unsupervised*). Figure S3 contains the resulting 2D MDS visualization.

**Figure S3**. 2D MDS visualization of the data based on pairwise Gower distances. Every point corresponds to a single patient. Points are marked and colored according to patients’ diagnoses. **Orange** denotes the patients labeled “appendicitis”, **blue** those labeled “non-appendicitis”, and “**?**” those labeled “appendicitis” without histological confirmation.

Every point in this plot corresponds to a patient. **Orange** denotes the patients labeled “appendicitis”, **blue** those labeled “non-appendicitis”, and “**?**” those labeled “appendicitis” without histological confirmation. Observe that the patients labeled “appendicitis” without histological confirmation (“**?**”) appear to fit well within the distribution of histologically confirmed cases (“**x**”). Of course, many of these points lie on the borderline between the classes. However, there is also a substantial number of ‘borderline’ histologically confirmed cases. In general, the data, as visualized by MDS, feature distinct interclass separability. Thus labeling seems to agree with variability in predictor variable distribution.

We also examined the certainty of the RF classifier given by the maximum predicted class probability. In Figure S4, data points are colored and sized according to the model’s certainty. **Blue** represents the data points the RF is most certain about, **orange** those that it is most *uncertain* about, while “**?**” represents unconfirmed appendicitis. Observe that for many histologically unconfirmed appendicitis cases the model is uncertain, likewise for many ‘borderline’ patients labeled “non-appendicitis”, which were not histologically confirmed either. In summary the RF model does not appear overconfident about patients with an unconfirmed diagnosis, which is reassuring.


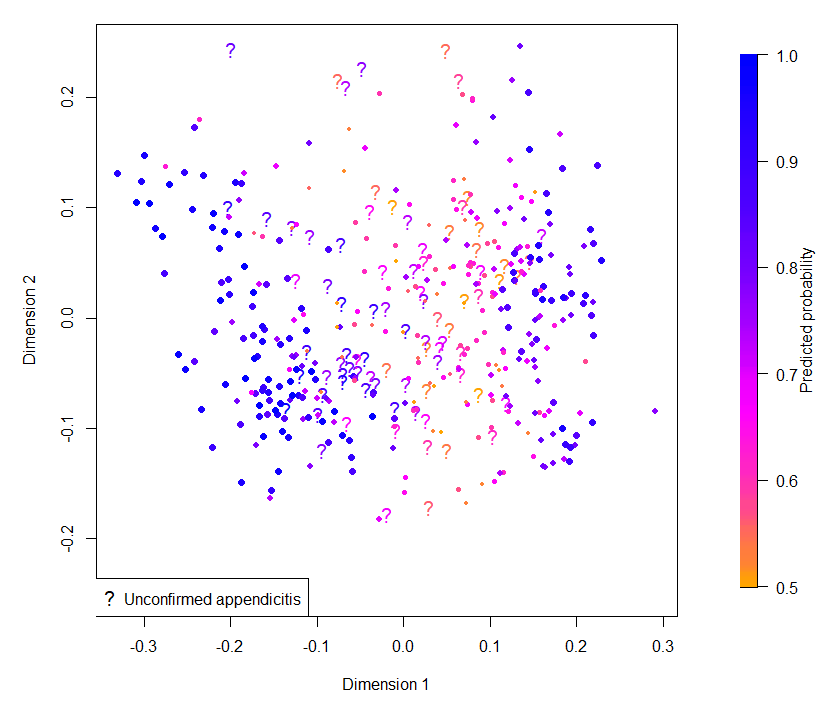


**Figure S4**. 2D MDS visualization of the data based on pairwise Gower distances. Every point corresponds to a single patient. Points are sized and colored according to the probability of the category (“appendicitis” or “no appendicitis”) as predicted by the random forest (RF) model. **Blue** represents the data points the RF is most certain about, **orange** those that it is most *uncertain* about, while “**?**” represents unconfirmed appendicitis.

In addition, we performed quantitative comparisons between two groups of patients: (i) those with histologically confirmed *uncomplicated* appendicitis and (ii) those with histologically unconfirmed appendicitis. As with the statistical analysis in the paper (see Table S3), statistical tests were performed for the differences in distributions of predictor variables; $p$-values were adjusted using Hommel’s method. The only predictors featuring significant differences ($\alpha=0.05$) across the two groups were peritonitis, irregular appendix layers, appendix diameter, WBC count, target sign, visibility of appendix in US, and neutrophil percentage. These are similar to the differences found for conservative and surgical management groups (Tables S4 and S5). Note that there were no significant differences in such important features as AS, PAS, rebound tenderness, body temperature, CRP etc. which differentiate appendicitis from non-appendicitis groups.

In summary, patient groups with histologically confirmed appendicitis and histologically unconfirmed appendicitis appear quite similar in feature distribution. In our view, this suggests that the labeling used for training and testing the models is sensible. We also observed that the RF classifier is quite uncertain about unconfirmed cases and is unlikely to be overfitting.
